# Single-Cell Characterization of *in vitro* Migration and Interaction Dynamics of T Cells Expanded with IL-2 and IL-7

**DOI:** 10.3389/fimmu.2015.00196

**Published:** 2015-04-28

**Authors:** Johanna Tauriainen, Karin Gustafsson, Mårten Göthlin, Jens Gertow, Marcus Buggert, Thomas W. Frisk, Annika C. Karlsson, Michael Uhlin, Björn Önfelt

**Affiliations:** ^1^Department of Laboratory Medicine, Division of Clinical Microbiology, Karolinska Institutet, Stockholm, Sweden; ^2^Science for Life Laboratory, Department of Applied Physics, KTH Royal Institute of Technology, Stockholm, Sweden; ^3^Center for Allogeneic Stem Cell Transplantation, Karolinska University Hospital Huddinge, Stockholm, Sweden; ^4^Department of Oncology and Pathology, Karolinska Institutet, Stockholm, Sweden; ^5^Department of Microbiology, Tumor and Cell Biology, Karolinska Institutet, Stockholm, Sweden

**Keywords:** T cell, IL-2, IL-7, microscopy, fluorescence, microchip, single-cell analysis, live-cell imaging

## Abstract

T cells are pivotal in the immune defense against cancers and infectious agents. To mount an effector response against cancer cells, T cells need to migrate to the cancer-site, engage in contacts with cancer cells, and perform their effector functions. Adoptive T cell therapy is an effective strategy as treatment of complications such as relapse or opportunistic infections after hematopoietic stem cell transplantations. This requires a sufficient amount of cells that are able to expand and respond to tumor or viral antigens. The cytokines interleukin (IL)-2 and IL-7 drive T cell differentiation, proliferation, and survival and are commonly used to expand T cells *ex vivo*. Here, we have used microchip-based live-cell imaging to follow the migration of individual T cells, their interactions with allogeneic monocytes, cell division, and apoptosis for extended periods of time; something that cannot be achieved by commonly used methods. Our data indicate that cells grown in IL-7 + IL-2 had similar migration and contact dynamics as cells grown in IL-2 alone. However, the addition of IL-7 decreased cell death creating a more viable cell population, which should be beneficial when preparing cells for immunotherapy.

## Introduction

T cells have been used in several adoptive cellular immunotherapies, which have shown promise as treatment for various cancers and infectious agents (1–4). The quality, the degree of activation, and the T cell profile are of central importance for clinical efficacy. Numerous *in vitro* T cell activation and expansion techniques have been described in previous studies, including use of anti-CD3- and anti-CD28-coated super paramagnetic beads and interleukin (IL)-2, in order to achieve high enough numbers of cells to be used clinically (5, 6). In addition, cytokines represent a polarizing signal that drives the development of recently activated, naïve CD4^+^, and CD8^+^ T cells toward various effector subsets (7–11). Accordingly, T cell expansion can be further propagated and controlled by the addition of various cytokines. The T cell growth factor IL-2 has well-documented effects on T cells from both *in vitro* models (12) and clinical trials (13–17). However, IL-2 administration has been shown to alter the homeostasis and increase the amount of CD4^+^CD25^hi^Foxp3^+^ regulatory T cells (T regs) in cancer patients dampening the desired response (18). In contrast, patients with metastatic cancers receiving IL-7 therapy showed a decrease of regulatory T cells and increases in CD4^+^ and CD8^+^ T cells (19). IL-7 has also been shown to enhance T cell proliferation, reduce activation-induced apoptosis and increase TCR diversity (20, 21).

A new fully glycosylated recombinant human (rh) IL-7 (Cyt107) was recently used in a clinical phase 1 study to enhance T-cell recovery after allogeneic stem cell transplantation (22). As previously reported, the treatment was shown to be well tolerated and safe (19, 22–27). Moreover, it has been shown that the combination of IL-2 and IL-7 can be used to modulate the proliferation and Fas-mediated cell death of distinct T cell subsets (28). Triggered by these observations, we set out to compare phenotypic and functional properties of T cells expanded in presence of anti-CD3- and anti-CD28-coated beads and IL-2 with or without the addition of rhIL-7. Hitherto, most of the *in vitro* characterization of expanded T cells is based on data from phenotype classification and cytokine profiles of T cells. Here, we have used a recently developed microchip-based approach (29–31) where we were able to follow the motility and cell–cell interaction patterns of individual T cells for hours in co-culture with allogeneic target cells.

## Materials and Methods

### Cell culture

Peripheral blood mononuclear cells (PBMC) were isolated from whole blood from 12 anonymous healthy donors using density gradient centrifugation (Lymphoprep, Fresenius Kabi Norge AS). According to local regulations, no ethical permit was required for anonymous blood donors. T cells were isolated from PBMC by use of paramagnetic beads coated with anti-CD3 and anti-CD28 antibodies (Dynabeads, Life Technologies, Grand Island, NY, USA) according to the manufacturer’s protocol. The isolated cells were expanded for 7 days together with the anti-CD3 and anti-CD28 beads in RPMI-1640 (Gibco, Life Technologies) containing 5% Human AB serum (Department of transfusion Medicine at Karolinska University Hospital, Huddinge), 100 U/mL Penicillin G, 100 μg/mL Streptomycin (Gibco, Life Technologies), and 2 mM l-glutamine (Sigma Aldrich Inc., St Louis, MO, USA). The cells were divided into two flasks, either with 100 IU/mL IL-2 (PeproTech, Rocky Hill, NJ, USA) or with a combination of 100 IU/mL IL-2 and 0.5 ng/mL rhIL-7 (Cyt107, Cytheris). Cells were cultured at 37°C, 5% CO_2_ and kept at a concentration of less than 3 × 10^5^ cells/mL. After 7 days of expansion, T cells were harvested and beads were removed from the cells by magnetic separation. Allogeneic monocytes were isolated from PBMC at the day of the experiment by allowing them to adhere to the bottom of a six-well plate. The non-adherent cells were removed and the adherent cells were mechanically detached from the wells before labeling and seeding in microwells. Allogeneic monocytes were chosen in order to stimulate interaction between T cells and target cells.

### Cell labeling

1 × 10^6^ cells were washed three times in RPMI-1640 and then stained with 0.5 μM Calcein Green AM (target cells) or 0.64 μM Calcein Red–Orange AM (T cells) (both dyes from Invitrogen, Carlsbad, CA, USA). Staining solutions were prepared with RPMI-1640 as solvent and added directly to the cell pellets, which were re-suspended and incubated for 10 min at 37°C. After staining, cells were washed three times in RPMI-1640 and used for experiments.

### Microchip

The microchip was prepared as described earlier (29). Briefly, the microchip was sterilized in ethanol and all traces of ethanol were removed by washing the chip in PBS after which the holder and chip were assembled. To enable imaging of two conditions simultaneously, the microchip was divided into two basins, one with IL-2 medium and the other with IL-2 + IL-7 medium by use of a polydimethylsiloxane (PDMS) gasket. Fluorescently labeled allogeneic target cells were added to each basin to a desired density (≈60 cells/well) and excess cells were removed by changing the medium in the chip. The target cells were then allowed to adhere for 1 h after which Calcein Red–Orange AM-labeled T cells from either the IL-2 only or IL-2 + IL-7 cultures were added to the chip (≈50 T cells/well) and were allowed to settle by sedimentation.

### Microscopy

Fluorescence and transmitted light images were obtained with an Olympus IX81-inverted confocal fluorescence microscope equipped with an environmental chamber keeping the cells at 37°C, 5% CO_2_. A motorized stage enabled automatic collection of images from selected parts of the microchip. For each experiment, four wells (two per condition) were imaged every 2 min for a period of 7 h with a 20× objective. The presented data are from three independent experiments with at total of 219 (IL-2) and 175 (IL-2 + IL-7) T cells, respectively.

### Image analysis

Images were analyzed with ImageJ software (US National Institutes of Health, Bethesda, MD, USA). Each T cell was manually tracked and interactions with target cells were scored. Interactions with target cells lasting at least two time points (4 min) were defined as a T cell contact with a target cell. Cell death was defined as visual signs of death in the transmitted light image, usually seen as blebbing or swelling of the cell membrane and/or leakage of the fluorescent dye. Cells that could not be followed for at least 60 min were excluded from the analysis.

### Immunophenotyping

After 7 days of expansion, T cells were harvested and immunophenotyping was performed. T cells were incubated with the following monoclonal antibodies: fluorescein isothiocyanate (FITC)-, phycoerythrin (PE)-, PE-cyanine 7 (PE-Cy7)-, and V450-labeled anti-CD3; allophycocyanin (APC)-, and Alexa Fluor 700-labeled anti-CD4; Peridinin-chloropyll-protein complex (PerCP)-labeled anti-7-Aminoactinomycin D (7-AAD); FITC-labeled anti-CD28; FITC-labeled anti-CD69; FITC-labeled anti-CD94; FITC-labeled anti-CD56; FITC-labeled anti-TCRαβ; FITC-labeled anti-CD95; APC-labeled anti-CD45RO; PE-Cy7-labeled anti-CCR7; FITC-labeled anti-CD25; APC-labeled Annexin V (all from BD Bioscience, Franklin Lakes, NJ, USA); FITC-labeled anti-TCRγδ (Becman Coulter, Fullerton, CA, USA); APC-Alexa Fluor 700-labeled anti-CD127 (Beckman Coulter, Fullerton, CA, USA); Pacific Orange-labeled anti-CD8 (Invitrogen); PE-labeled anti-CD39 (BD Bioscience, Franklin lakes, NJ, USA) for 15 min and washed before flow cytometry (FACS Aria flow cytometer, BD Biosciences) and analysis (FlowJo software, Tree Star, Inc., Ashland, OR, USA). T cell death was assessed by flow cytometry staining for 7-AAD and Annexin V after 7 days culture with beads and cytokines followed by a brief (6 h) period of stimulation with peptide mix spanning the cytomegalovirus (CMV) protein pp65.

### Statistical analysis

Statistical analysis was performed with Prism (GraphPad, San Diego, CA, USA) or Matlab (MathWorks, MA, USA) using the Mann–Whitney *U* test to evaluate migration and bulk expansion data, the Wilcoxon matched-pairs signed rank test to compare fold expansion data and Chi-square test to evaluate cell–cell contacts, cell division, and cell death. In the images, stars have been used to denote significance levels according to; * (*p* < 0.05), ** (*p* < 0.01) and “n.s.” refers to no statistical significance. Box-and-whisker plots indicate second and third quartiles separated by the median (inside the box) and whiskers show minimum and maximum values.

## Results

### Expansion of T-cells with IL-2 or IL-2 + IL-7

**Figure 1:**
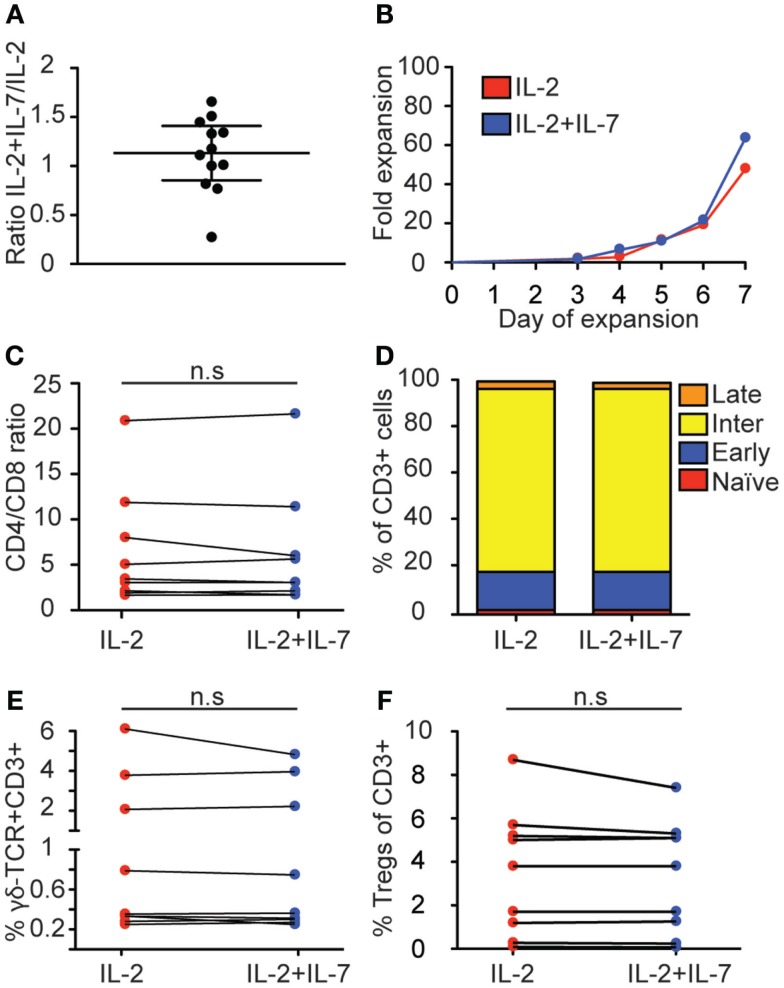
**Effect of IL-7 on T cell expansion and phenotype**. T cells were positively selected and cultured for 7 days with anti-CD3- and anti-CD28-coated beads and IL-2 + IL-7 or IL-2 alone, after which the cells were counted and phenotyped by flow cytometry and the expressions of CD4, CD8, CCR7, CD45RO, γδ-TCR, CD25, and CD39 were compared between the two culture conditions. **(A)** Relative T cell expansion displayed as ratio of fold increase in IL-2 + IL-7 over IL-2. **(B)** Kinetics of fold expansion for one representative donor. This donor gave median fold expansion after 7 days for both culture conditions. **(C)** CD4/CD8 ratio. **(D)** Distribution of memory subsets. T cell maturation profile from least mature to most mature was defined as: CCR7^+^CD45RO^−^ (naïve, red); CCR7^+^CD45RO^+^ (early-differentiated, blue); CCR7^−^CD45RO^+^ (intermediate-differentiated, yellow); CCR7^−^CD45RO^−^ (late-differentiated, orange). **(E)** Percentage of CD3^+^CD4^−^CD8^−^T cells expressing γδ-TCR. **(F)** Percentage of regulatory T cells, defined as CD3^+^CD4^+^CD127^−^CD25^+^CD39^+^. Flow cytometry data are based on data from 9 individual donors, and cell expansion data is based on 12 individual experiments. Lines connect cultures from the same donor, horizontal lines in **(A)** depict the median and IQR. Mann–Whitney tests were performed to evaluate significance between the groups.

We set out to test if the addition of a fully glycosylated form of rhIL-7 (Cyt107) could improve the yield and quality of T cells compared to an expansion protocol with only IL-2. This form of IL-7 was recently used in a clinical setting to successfully enhance T cell recovery after allogeneic stem cell transplantation (22). T cells were positively selected from PBMC with anti-CD3 and anti-CD28 paramagnetic beads and cultured in medium containing IL-2 alone or a combination of IL-2 and IL-7. Cells were cultured for 7 days and kept at a concentration of no more than 3 × 10^5^ cells/mL. The yield varied significantly between individual expansions (*n* = 12) for both culturing conditions; the combination of IL-2 and IL-7 showed the fold-increase range 12–193 with median 51 while IL-2 alone gave a range of 10–183 and median 50. Addition of IL-7 led to increased cell numbers in 7 out of 12 expansions, while 2 expansions showed similar numbers for both conditions and three expansions gave a higher fold expansion for IL-2 alone, shown as the ratio of fold increase in IL-2 + IL-7 over IL-2 at day 7 for each experiment (Figure 1A); however, no statistically significant difference was found (*p* = *0.52*, Wilcoxon matched-pairs signed rank test). Kinetic analysis of T cell proliferation showed that cell numbers increased exponentially until the end of culture at 7 days for both conditions (Figure 1B; Figure S1 in Supplementary Material). Thus, both culture conditions stimulated substantial T cell proliferation displaying large variations between individual blood donors and addition of IL-7 had no statistically significant effect on the total T cell yield.

### IL-7 did not affect expression of phenotypic markers of peripheral blood-derived T cells

When T cells are activated and expanded *in vitro*, their phenotype and function change. In order to test if addition of IL-7 had an affect over IL-2 on the T cell phenotype, anti-CD3- and anti-CD28-activated peripheral blood-derived T cells cultured in IL-2 + IL-7 or IL-2 alone for 7 days were analyzed by multi-color flow cytometry (see Figure S2 in Supplementary Material for gating strategy). First, the CD4/CD8 ratios were compared, displaying significant variation between individual donors but no statistically significant difference correlated to the addition of IL-7 was detected (medians were 3.1 and 3.5 for IL-2 and IL-2 + IL-7, respectively) (Figure 1C).

Next, we tested the effect of IL-7 on differentiation. Stages of differentiation were defined based on expression of CCR7 and CD45RO. Naïve T cells were defined as CCR7^+^CD45RO^−^; early-differentiated T cells as CCR7^+^CD45RO^+^; intermediate-differentiated T cells as CCR7^−^CD45RO^+^; and late-differentiated as CCR7^−^CD45RO^−^ (32, 33). No significant differences were seen for memory T cell subsets in the CD3^+^ T cell compartment when T cells were expanded in the presence or absence of IL-7 (Figure 1D). In both conditions, intermediate-differentiated T cells were the most frequently observed population followed by early-differentiated T cells, late-differentiated T cells, and naïve T cells.

Furthermore, the percentage of γδ-T cells was compared between cultures with or without IL-7. No difference in CD3^+^CD4^−^CD8^−^T cells expressing γδ-TCR was observed (Figure 1E). It has been described that treatment with IL-2 may increase the amount of regulatory T cells in cancer patients (18), while IL-7 may have opposite effects (19). Therefore, the proportions of T regs (defined as CD3^+^CD4^+^CD127^−^CD25^+^CD39^+^) were compared between the two culture conditions but no difference was detected (Figure 1F). Thus, these results show that for T cells expanded *in vitro* from peripheral blood the addition of IL-7 had little effect on CD4/CD8 ratio, memory status and frequency of regulatory T cells.

### Addition of IL-7 does not influence T cell migration and interaction with target cells

**Figure 2:**
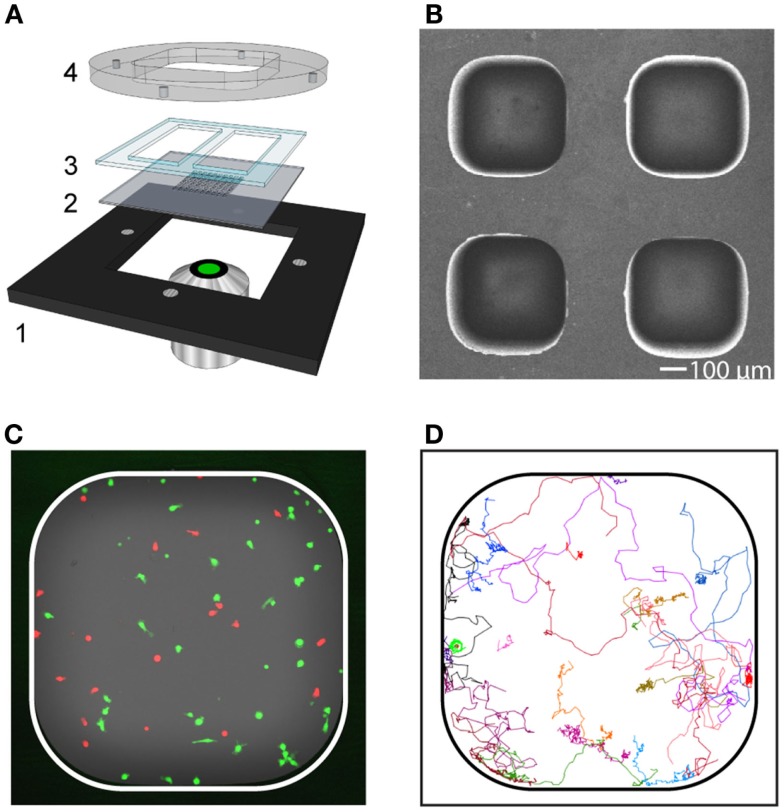
**Long-term live-cell imaging in microwells for characterization of migration and contact dynamics**. **(A)** Schematic picture of the device consisting of a metal holder (1), multi-well silicon-glass microchip (2), PDMS gasket (3), and lid (4). The lid was held tightly against the metal holder by magnets. The PDMS gasket was cut to create two separate basins (one per condition), each containing approximately 30 microwells. **(B)** Scanning electron microscopy image showing a subsection of the microchip. The dimension of the microwell bottom was 450 × 450 μm^2^ and the well depth was 300 μm. **(C)** Fluorescence image of a microwell containing T cells (red) and target cells (green). **(D)** Trajectories from T cells (*n* = 23) followed in a representative time-lapse sequence.

To investigate the impact that the expansion protocols had on T cell behavior, a microchip-based live-cell imaging assay was used (Figure 2). With this approach, time-lapse imaging enabled a detailed comparison of migration and interaction properties of individual T cells over time between cultures expanded with or without IL-7. T cells collected after both culture conditions were fluorescently stained and seeded to separate microwells loaded with allogeneic monocytes. Two to four microwells for each experimental condition were selected and automatically imaged periodically with a confocal microscope equipped with a motorized stage creating up to four individual time-lapse movies from each experiment. Individual T cells were tracked and their interactions with allogeneic target cells recorded.

**Figure 3:**
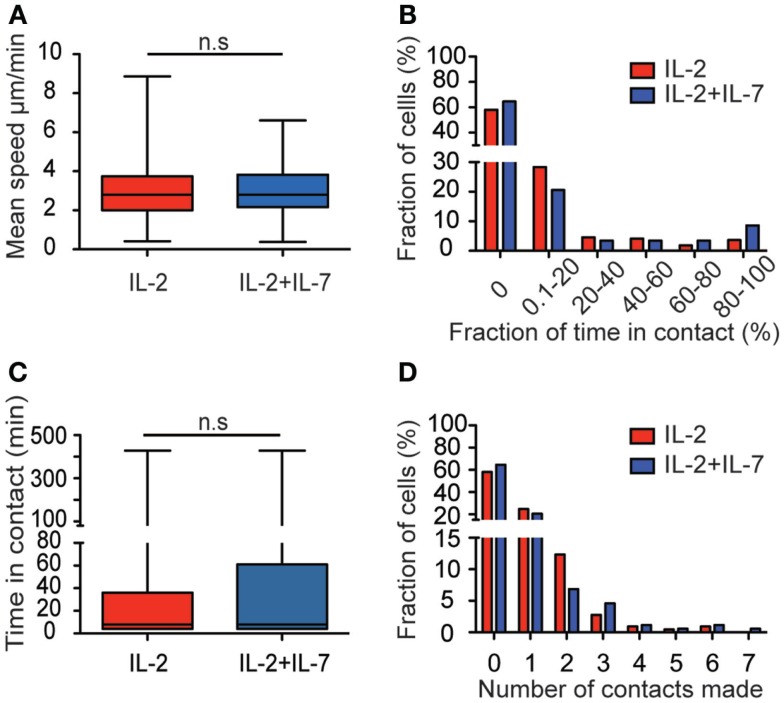
**Addition of IL-7 does not influence T cell migration and target cell interaction dynamics**. **(A)** Box plot of T cell migration speed for the two conditions. The mean speed of T cells cultured in IL-2 (red, *n* = 219, median 2.8 μm/min), and T cells cultured in IL-2 + IL-7 (blue, *n* = 175, median 2.8 μm/min). **(B)** The fraction of time (%) spent in contact with target cells shown for cells cultured in IL-2 (red, *n* = 219) or IL-2 + IL-7 (blue, *n* = 175) during the time interval they were followed. **(C)** Boxplot of the duration of all individual contacts scored between T and target cell during the 7-h assay for the two conditions (*n* = 152 for IL-2 and *n* = 125 for IL-2 + IL-7). Median times per contact were 8 min for both conditions. **(D)** Histogram showing the number of contacts made with target cells by individual T cells (%) during the 7-h assay for cells cultured in IL-2 (red bars, *n* = 219 cells) or in IL-2 + IL-7 (blue bars, *n* = 175 cells). Mann–Whitney tests were performed to compare mean speed, fraction of time in contact, time spent in each individual contact, and number of contacts made by each T cell between the two culture conditions.

The median migration speeds for T cells were 2.8 μm/min for both culture conditions (Figure 3A). The percentage of time a T cell spent in contact with target cells during the entire assay of 7 h was also assessed (Figure 3B). Among the T cells that made contact with target cells, the vast majority spent a low fraction of time (<20%) in contact while others remained in contact during the whole assay (Figure 3B). Evaluation of the duration of all individual contacts between T cells and target cells during the assay showed that most contacts were brief, with a median of 8 min for both culture conditions (Figure 3C). However, also significantly longer contacts were observed, some of them lasting up to 7 h. For cells cultured in IL-2 only, 42.5% made contact with a target cell during the assay whereas 35.4% of the cells cultured in a combination of IL-2 + IL-7 made contacts (Figure 3D). Individual T cells were observed to make contact with up to seven individual target cells during the 7-h assay. No statistically significant difference was observed in the number of contacts made by T cell from the two populations. The data in Figure 3 are pooled from three independent experiments, which are presented separately in Figure S3 in Supplementary Material.

**Figure 4:**
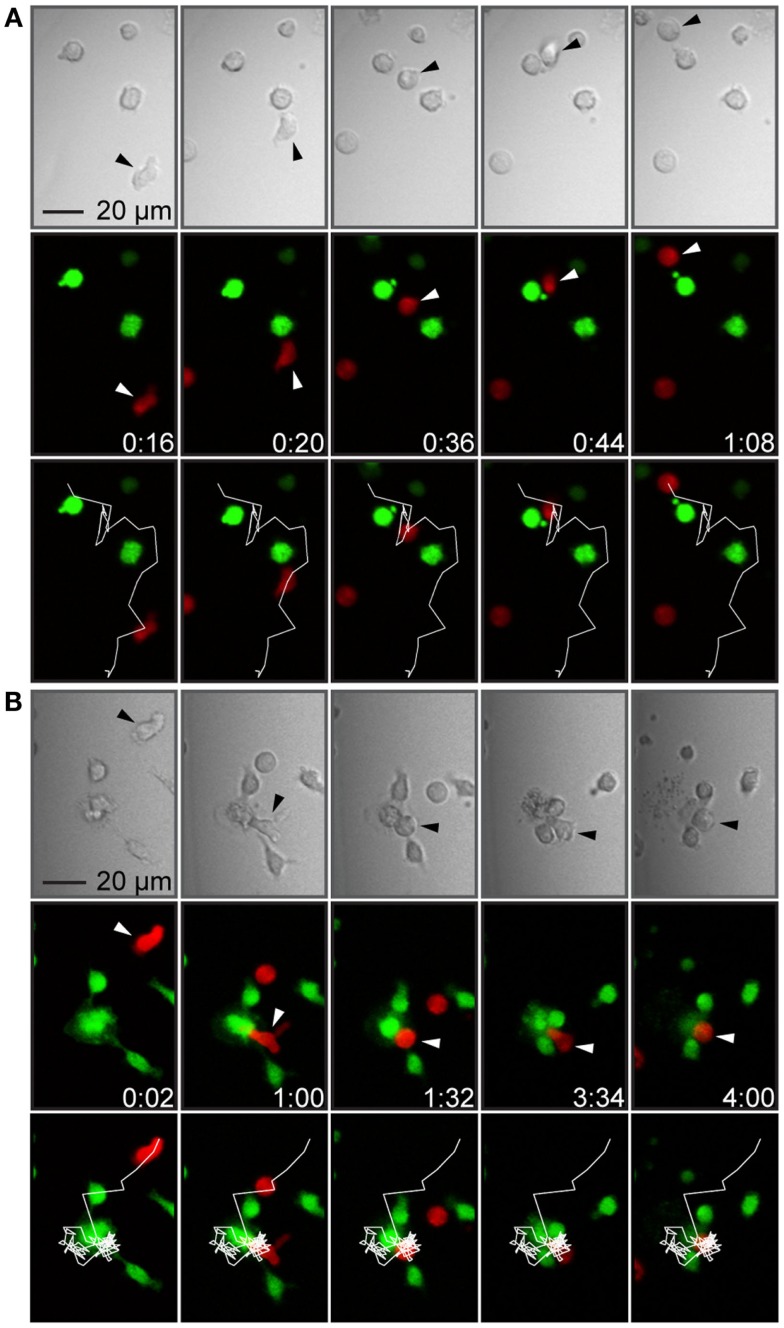
**T cell–target cell interactions were brief or more stable, sometimes leading to target cell death**. **(A)** Time-lapse sequence showing a T cell forming a brief contact with a target cell. The panels show transmitted light (top), fluorescence showing T cells in red and target cells in green (middle) and fluorescence overlaid with the T cell trajectory (white line, bottom). A T cell (marked by arrowheads) migrated toward a target cell (frame 1), formed a contact with the target cell (frame 2), continued migrating while attached to the target cells, scanning the target cell surface (frames 3 and 4), and finally detached without forming a stable conjugate or killing the target cell (frame 5). **(B)** Time-lapse sequence showing T cell-mediated target cell death. Panels as in **(A)**. A T cell (marked by arrowheads) migrated with an elongated shape (frame 1), made a contact with a target cell within a cluster of three cells (frame 2), rounded up, and formed a conjugate (frame 3), which was followed by target cell death seen as a decreased green fluorescence intensity and a cloud of cellular debris in the transmitted light channel (frames 4 and 5). Indicated times are hours:minutes and scale bar represent 20 μm. Images have been resampled and brightness and contrast altered to improve visibility.

When T cells made contact with target cells, the most common observation made was that the T cells scanned the surface of the target cell, detached, and continued migrating (Figure 4A). Although the vast majority of T cell–target cell interactions did not result in T cell-mediated target cell killing, a few killing events could be observed in the time-lapse movies (Figure 4B). In these events, target cells engaged in conjugates with T cells were observed to change their morphology manifested as membrane blebbing, swelling, and bursting correlated with decreased fluorescence intensity due to leakage of calcein. However, too few events were observed in order to assess any difference between culture conditions.

### Addition of IL-7 decreases cell death but shows no effect on the rate of mitosis

**Figure 5:**
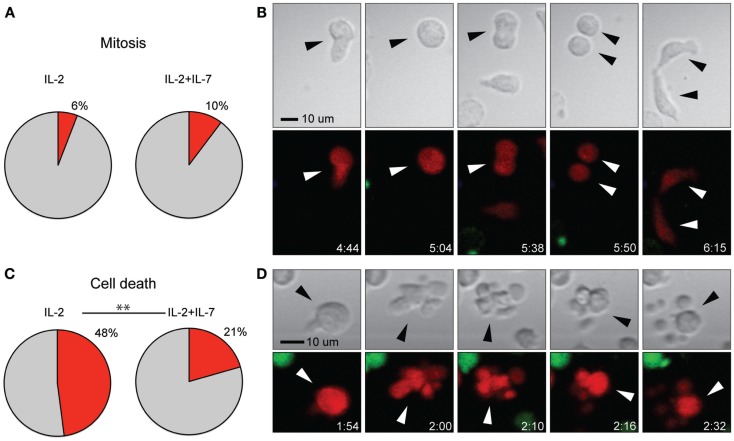
**Addition of IL-7 had no effect on rate of T cell mitosis but decreased cell death**. **(A)** Percentage of T cells undergoing mitosis during the 7-h assay for the two conditions. **(B)** Time-lapse imaging data of cell division shown in transmission (top) and fluorescence (bottom). A T cell (red fluorescence) migrated with a typically elongated, migratory shape (frame 1) and stopped, enlarged, and rounded up (frame 2), this interphase was followed by mitosis where the cells split into two daughter cells (frames 3 and 4) and finally cytokinesis (frame 5), where the new cells were divided. Indicated times are hours:minutes and the scale bar represents 10 μm. **(C)** Percentage of T cells dying during the 7-h assay for the two conditions (*p* < 0.005). **(D)** Time-lapse imaging data of T cell death shown in transmission (top), fluorescence (bottom). A migrating T cell (red fluorescence) stopped and rounded up (frame 1), followed by cell death seen as membrane blebbing in the transmitted light image (top) and decreased red fluorescence intensity (bottom) (frames 2–5). Indicated times are hours:minutes and scale bar represents 10 μm. Chi-squared test was used to compare the rate of cell death and mitosis between the two culture conditions. The data in **(A,C)** are based on three individual experiments (IL-2 *n* = 219, IL-2 + IL-7 *n* = 175). Images have been resampled and brightness and contrast altered to improve visibility.

Stimulation with anti-CD3- and anti-CD28-coated paramagnetic beads efficiently induced T cell proliferation in both culture conditions tested. However, for T cells used in immunotherapy, it is of central importance that expanded cells are of high quality and retain a proliferative capacity after injection. Therefore, the frequency of cell division (Figures 5A,B) in relation to cell death (Figures 5C,D) was assessed from the time-lapse movies. While no difference in cell division was observed between the two culture conditions, it was found that the rate of cell death was higher for T cells expanded in IL-2 alone (*p* < 0.005, Chi-square test) (Figure 5). The data in Figure 5 are pooled from three independent experiments, which are presented separately in Figure S3J,K in Supplementary Material. However, when cell death was measured by flow cytometry, staining for the markers 7-AAD and Annexin V after 7 days of culture followed by a brief period of peptide stimulation, no differences in the rate of cell death were observed between the two culture conditions, and the percentages of dead cells were low (median 4.9% for IL-2 vs. 4.1% for IL-2 + IL-7, Figure S2F in Supplementary Material). Thus, taken together our data indicate that any beneficial effect for T cell yield given by addition of IL-7 at these cytokine concentrations is not due to increased proliferation but rather increased tolerance to stress leading to better survival.

## Discussion

For patients where T cell immunotherapy may be considered, the quality and number of lymphocytes to be infused are of central importance for the clinical outcome. Stable and reproducible T cell culture protocols in combination with efficient evaluation methods are needed. Progress has been made in designing strategies to analyze both the frequency of specific T cells and their ability to produce cytokines at a single-cell level. New multiparametric flow cytometry assays combining phenotypic and functional characterization of T cell populations are now used frequently due to their ability to measure multiple cytokines and provide detailed information about phenotypically different subpopulations. However, these methods do not reveal dynamical properties such as migration and formation of immune synapses, properties that are central for a functioning T cell response.

Here, we use a recently developed microchip-based, live-cell imaging approach, with single-cell resolution (29–31). This assay enables detailed characterization of individual T cells’ migration behavior and contact history over time as well as information about cell division and apoptosis. With this method, it is also possible to detect rare events occurring in small subpopulations of tumor-reactive T cell clones. Together with other recently developed microchip methods, e.g., microengraving (34, 35), this tool could be valuable for how to select and expand cells for therapy.

A central issue for adoptive T cell-based immunotherapies is how to generate enough functional cells in adequate time. In the present study, efficient T cell expansion was obtained within a week by co-culturing peripheral blood-derived T cells with artificial APCs, i.e., anti-CD3- and anti-CD28-coated beads. Cytokines were added to induce further activation and modulation of the expanding T cell population. IL-2 is the most frequently used cytokine for this purpose as it is a potent T cell mitogenic cytokine and essential for the growth, proliferation, and differentiation of T cells. Generally, this type of T cell expansion protocol generates large numbers of polyclonal T cells within a relatively short time. However, IL-2 has been shown to be involved in the priming of mature T cells for activation-induced cell death by enhancing expression of pro-apoptotic molecules such as FasL, and to suppress apoptosis inhibitors of Fas signaling (36). A possible drawback with this type of protocol is therefore the risk for massive apoptosis after injection due to sudden withdrawal of cytokines (37).

In an attempt to generate an expanded T cell population with better capacity to survive, IL-7 was added to the T cell cultures. IL-7 has also been shown to enhance T cell proliferation, but in contrast to IL-2 it has been reported to also reduce activation-induced apoptosis (38). In the present study, we used a fully glycosylated form of IL-7 that has been shown in a clinical phase 1 study to successfully enhance T cell recovery after allogeneic HSCT (22). Our proliferation studies of bulk cultures indicated little or no beneficial effects from the addition of IL-7, and no phenotypic differences were detected between cells cultured with or without IL-7. This is not in line with our previous experiments from cord blood-derived T cells where an increased expansion was observed after the addition of IL-7 (39). In the earlier study, we also observed that the CD4/CD8 ratio was higher in cultures containing IL-7 and IL-2 compared to IL-2 alone (median 3.0 vs. 2.4). In the present study, we see a slightly higher CD4/CD8 ratio for T cells cultured in a combination of IL-2 + IL-7 compared to IL-2 only (median 3.5 vs. 3.1); however, this did not reach statistical significance. Berglund et al. used IL-7 produced in *E. coli*, with a glycosylation pattern differing from that found in eukaryotic cells, something that could possibly account for the differences observed. In addition, that study used CB-derived T cells, which are known to have a more naïve phenotype than T cells isolated from PBMC that were used in the present study, changing the response pattern to cytokine exposure.

The microchip-based time-lapse imaging assay was used to evaluate the behavior and survival capacity of the T cells at a time point (7 days) when cells expanded for T cell-based immunotherapy are normally injected into the patient. Most T cells making contact with target cells were observed to scan the surface of the target cell rather than forming stable immune synapses. This behavior is consistent with only a small fraction of the T cells being allospecific. T cell migration and contact dynamics were found to be similar under the two culturing conditions. However, we observed a significant decrease in T cell death in the imaging experiments with the addition of IL-7. This decrease was not reflected in the flow cytometry analysis using the markers 7-AAD and Annexin V for measuring cell death. A reason why we observe more cell death in the imaging experiments could be that some of the dying cells rapidly disintegrate, and while such death is detected by imaging it is not by flow cytometry where such cells are instead washed away in the cleaning steps. However, it is plausible that there were negative effects from the microchip assay with fluorescence labeling and repeated imaging, although similar protocols have proved functional and non-harmful for IL-2-activated natural killer cells (29, 30). It is possible that the T cells generated with these protocols are over-stimulated and easily die when separated from the anti-CD3 and anti-CD28 beads and/or handled in the lab. Either way, the cells could face similar stress during preparation and infusion in a clinical setting resulting in a large fraction of the cells dying shortly after infusion. Our imaging data indicate that the addition of IL-7 makes the T cells more resistant to stress.

In summary, there is a need for methods that can probe the condition of cells expanded for immunotherapy. The microchip-based assay presented here allows for assessment of cellular behavior that could be of clinical interest, including migration properties, interaction and killing of target cells, mitosis, or apoptosis. Since detection is at the single-cell level, it allows characterization also of rare clones within populations, clones that could have beneficial or detrimental clinical impact. Relatively little material is required to run the assay making it possible to study the behavior of cells from small clinical samples. Increased knowledge of the behavior and survival capacity of *in vitro* cultured lymphocytes aimed for treatment of various immunological issues opens for new immunotherapeutic perspectives with implications in areas such as transplantation medicine, autoimmune diseases, and HIV/AIDS research.

## Conflict of Interest Statement

The authors declare that the research was conducted in the absence of any commercial or financial relationships that could be construed as a potential conflict of interest.

## Supplementary Material

The Supplementary Material for this article can be found online at http://journal.frontiersin.org/article/10.3389/fimmu.2015.00196
