# Supplementary material for: Single-Cell Characterization of in vitro Migration and Interaction Dynamics of T Cells Expanded with IL-2 and IL-7
Source: Front Immunol. 2015 Apr 28;6:196. doi: 10.3389/fimmu.2015.00196 (PMC4412128; doi:10.3389/fimmu.2015.00196)
Supplement: Supplementary file 1 [file Data_Sheet_1.PDF]

Supplementary material

Single-cell characterization of in vitro migration and interaction dynamics of T cells expanded with IL-2 and IL-7

Johanna Tauriainen[1], Karin Gustafsson[2], Mårten Göthlin[2], Jens Gertow[3,4], Marcus Buggert[1], Thomas W Frisk[2], Annika C Karlsson[1], Michael Uhlin[3,4*], Björn Önfelt[2,5*]

[1]Department of Laboratory Medicine, Division of Clinical Microbiology, Karolinska Institutet, Stockholm, Sweden
[2]Science for Life Laboratory, Department of Applied Physics, KTH – Royal Institute of Technology, Stockholm, Sweden
[3]Center for Allogeneic Stem Cell Transplantation, Karolinska University Hospital Huddinge, Stockholm, Sweden
[4]Department of Oncology and Pathology, Karolinska Institutet, Stockholm, Sweden
[5]Department of Microbiology, Tumor and Cell Biology, Karolinska Institutet, Stockholm, Sweden

***Correspondence:** Björn Önfelt**,** Science for Life Laboratory, Tomtebodavägen 23A, 17165 Solna, Sweden**.**
bjorn.onfelt@ki.se

Michael Uhlin, Center for Allogeneic Stem Cell Transplantation (CAST) B87, Karolinska University Hospital Huddinge, 141 86 Stockholm
Michael.uhlin@ki.se

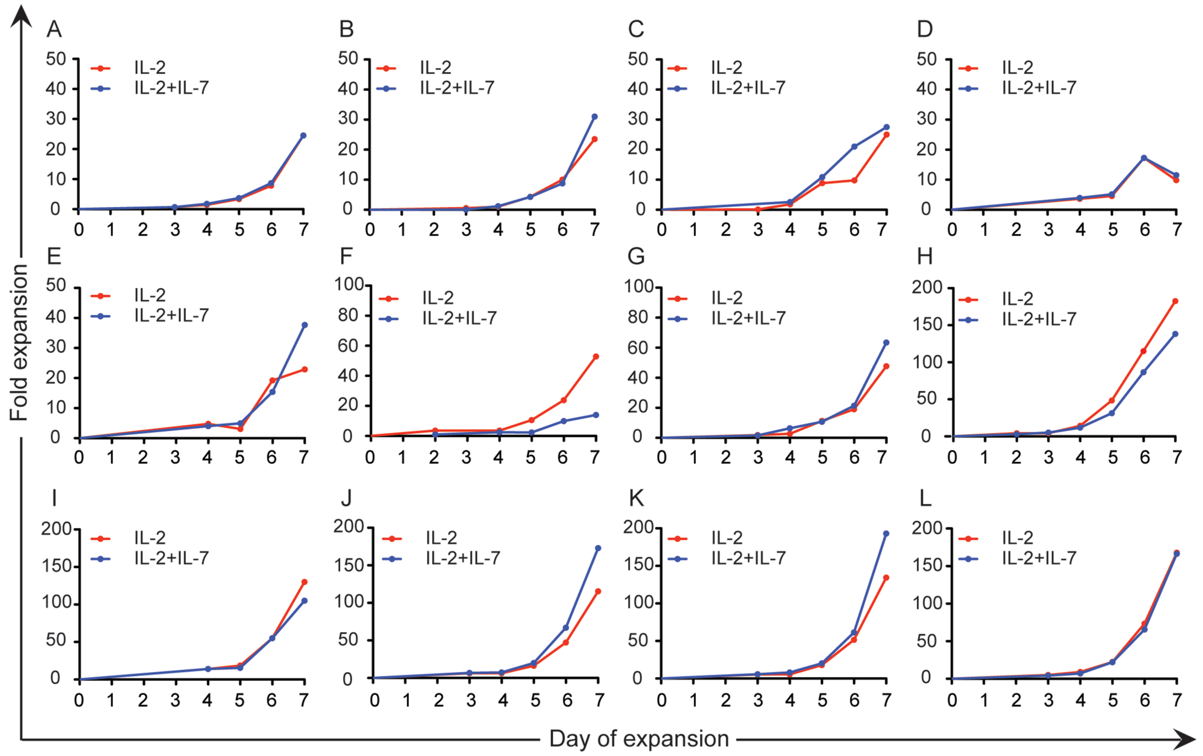

Figure S1. Fold expansion data for all individual T cell expansions. Graphs depict the fold expansion during the 7 day expansion for n=12 T cell expansions. A-E show expansions up to 50-fold, F-G show expansions up to 100-fold and H-L show expansions up to 200-fold.

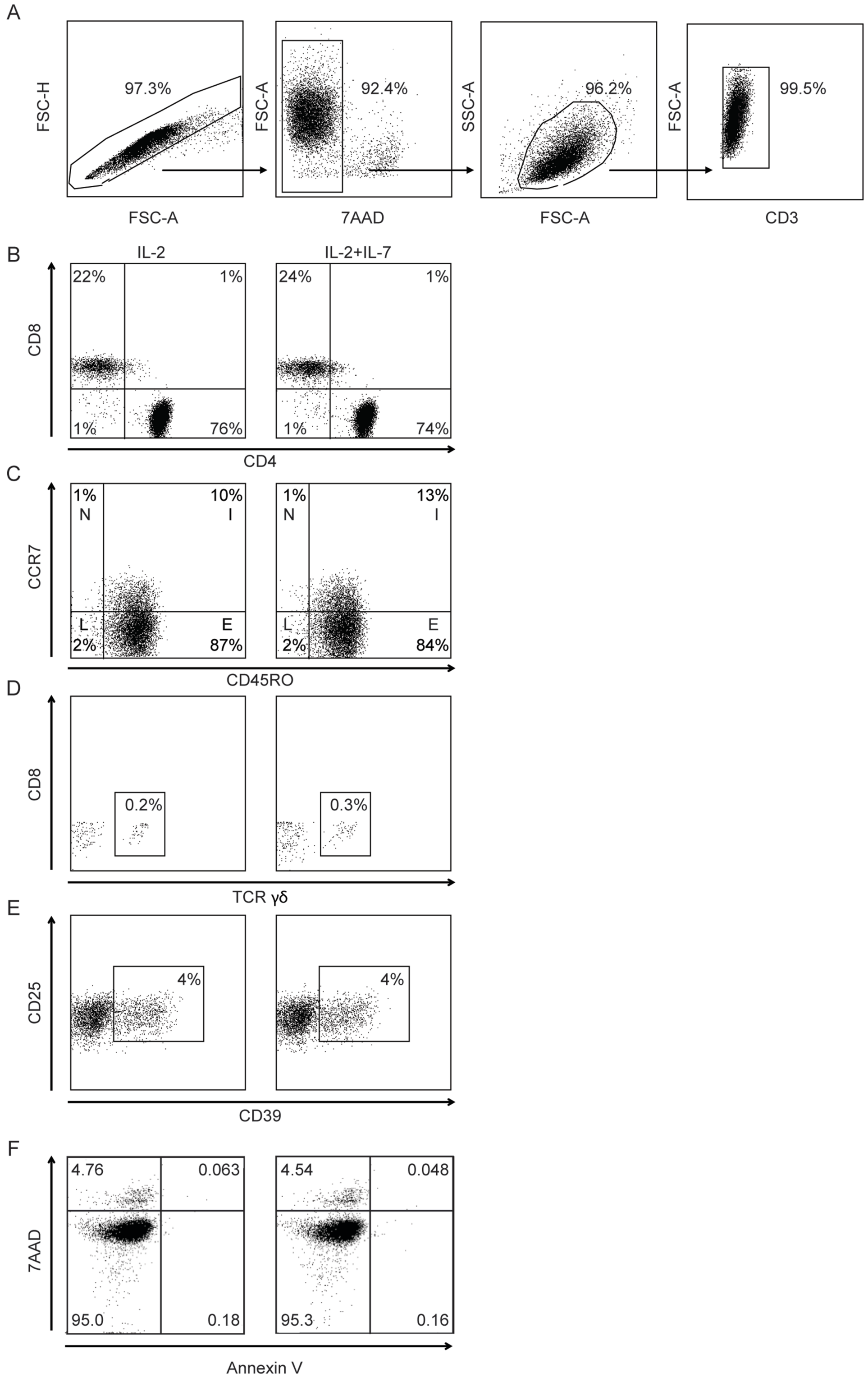

Figure S2. Gating strategy for flow cytometry-based characterization T cell phenotype. A. Gating strategy to distinguish live CD3+ lymphocytes. B. CD4/CD8 ratio (gated on CD3+ T cells). C. Differentiation status (N = Naïve, Early = early differentiated, Int = Intermediate differentiated, Late = late differentiated) (gated on CD3+ cells). D. γδ TCR+ CD3+ T cells (gated on CD3+CD4-CD8- cells). E. Regulatory T cells (gated on CD3+CD4+CD127- cells). F. Dead/apoptotic cells after a brief (6h) period of peptide stimulation (CMV pp65). Displayed FACS-plots are representative from nine independent donors except panel F that is representative from three donors. The respective markers used are indicated on the axes. For B, C and F gate statistics indicate the population frequency expressed as percentage of CD3+ cells.

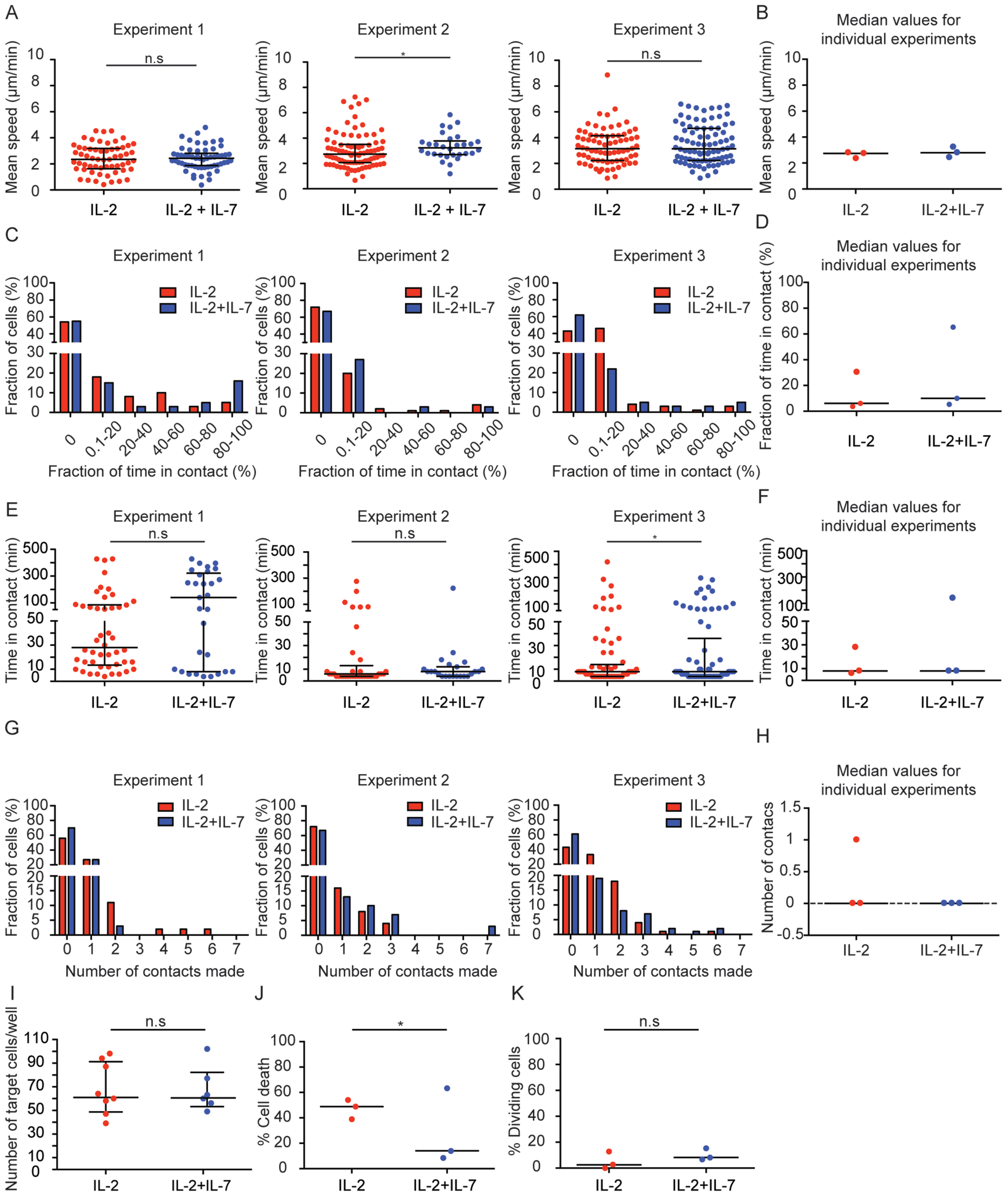

Figure S3. T cell migration and contact dynamics for individual T cell expansions. A. Mean speeds for T cells tracked for Experiment 1, 2 and 3 (* represents $p=0.04$). B. Median values from the experiments in (A). C. Histograms showing the distributions of fractions of time (%) spent in contact with target cells shown for the three individual experiments. D. The median values from the experiments in (C). E. Time (min) spent in individual contacts for cells from the three experiments (* represents $p=0.02$). F. The median values from the experiments in (E). G. Histograms showing the distributions of number of contacts made by individual cell for the three experiments. H. The median values from the experiments in (G). I. The number of live target cells at time point 1 for each well analyzed for IL-2 (red, n=8) and IL-2+IL-7 (blue, n=6). J. Fractions (%) of T cell dying during the assay for the three individual experiments. K. Fractions of T cells undergoing cell division during the time interval followed for the three individual experiments. The number of cells in the individual experiments where $n_1=59$, $n_2=86$ and $n_3=74$ for IL-2 and $n_1=59$, $n_2=30$ and $n_3=86$ for IL-2+IL-7. The Mann-Whitney test was used to compare results from the two culture conditions. Chi-squared test was used to compare the fraction of cell death and cell division. Horizontal bars represent median (panels A, B, D, E, F, H and I) with IQR (panels A, E and I), or mean with SEM (panels J and K).
